# Editorial

**Published:** 1838-06-06

**Authors:** 


### THE MEDICAL EXAMINER.

PHILADELPHIA, JUNE 6, 1838.

Dr. Drake, of the Cincinnati Medical College, announces that he is preparing for the press a work on the elements of Pathology. It is intended, he informs us, chiefly for circulation in the valley of the Mississippi, "as he does not anticipate for it a favourable reception East of the mountains"! Does the doctor, then, set no value on tramontane medical criticism? Can he hold no communion with any of the varieties of opinion that are current here? Or does he suppose, that, from sectional jealousy, his Atlantic brethren are unwilling to do justice to the merits of the West? We cannot impute to Dr. Drake a design to foist his writings into popularity, by fomenting provincial prejudices; we know and appreciate his devotion to the interests of his locality; but we did not expect from him the unphilosophic illiberality, that is evinced in the attempt, (if we are right in giving this construction to his language,) to isolate the valley of the Mississippi from the general republic of medical literature. We are especially adverse to such an isolation, in the case of an author, like Dr. Drake, from the productions of whose talents, the East and West, North and South, must assert their equal claims for edification.

